# Visual crowding: Double dissociation between orientation and brightness judgments

**DOI:** 10.1167/jov.23.5.7

**Published:** 2023-05-09

**Authors:** John Cass, Erik Van der Burg

**Affiliations:** 1 The MARCS Institute of Brain, Behaviour & Development, Western Sydney University, Sydney, Australia; 2 Brain and Cognition, University of Amsterdam, Amsterdam, Netherlands; 3 TNO, Human Factors, Soesterberg, Netherlands; 4 Department of Experimental and Applied Psychology, Vrije Universiteit, Amsterdam, Netherlands

**Keywords:** crowding, lightness/brightness perception, orientation

## Abstract

Peripherally presented objects are often more difficult to identify when located in cluttered visual environments than when presented in isolation, a phenomenon known as visual crowding. Crowding tends to be stronger when target and nearby flanking elements are composed of similar sets of features. This study investigates the extent to which target–flanker orientation and/or color similarity determines luminance and orientation performance across different tasks under identical stimulus conditions. Targets were near-vertical Gabor patches defined by modulating only the green component of the RGB display. Subjects performed both target luminance and orientation discrimination tasks in separate blocks while both flanker hue (green or red flankers) and orientation (vertical or horizontal flankers) were manipulated as a function of target–flanker separation. We find strong evidence for a double dissociation between task and the specific set of features by which target–flanker similarity is defined. Whereas luminance judgments were highly contingent upon target–flanker hue similarity, orientation judgments showed the inverse pattern, largely contingent upon flanker orientation. The magnitude of this double dissociation decreased with target–flanker separation, at a rate predicted by Bouma's law. This specific pattern of performance provides strong support for the idea that crowding operates independently for the most part within orientation and color domains. That luminance judgments are constrained by target–flanker flanker hue similarity and, to a far lesser extent, by target–flanker orientation similarity suggests that the neural mechanisms responsible for mediating perceived luminance are principally linked to those mediating stimulus hue independent of those mediating stimulus orientation.

## Introduction

Peripherally presented visual objects are often more difficult to identify than those closer to fixation. There are several reasons for this, all of which have neurogenic rather than optical causes. Principal of these are the precipitous reduction in cone receptor density (Curcio, Sloan, Kalina, & Hendrickson, 1990) combined with increasing postreceptoral neural convergence associated with increasing visual eccentricity. Both factors cause loss of spatial resolution, making objects in our peripheral vision appear blurred relative to foveal images (Drasdo, 1977; Levi & Klein, 1990; Levi & Waugh, 1994). Another major factor contributing to the loss of peripheral object recognition performance is *visual crowding*: the impaired ability to identify peripherally presented target objects due to the mere presence of neighboring flanking stimuli (a.k.a. clutter) (Korte, 1923). So profound, spatially extensive, and ubiquitous are the effects of visual crowding under natural viewing conditions (Wallis & Bex, 2012) that it has been described as the greatest constraint on visual object recognition (Pelli & Tillman, 2008).

The stimulus contingencies that produce visual crowding have been studied extensively (for reviews, see Pelli & Tillman, 2008; Strasburger, Rentschler, & Juttner, 2011; Whitney & Levi, 2011). Of these, two stimulus-based principles have been established that predict whether crowding is likely to occur for a given target stimulus. One of these principles, known as *critical spacing* or *Bouma's law*, predicts that target identification errors are more likely when flanking stimuli are located within a radius of the target equal to (and less than) approximately half the target's visual eccentricity (Bouma & Andriessen, 1970; Levi, 2008; Pelli & Tillman, 2008; Van der Burg, Olivers, & Cass, 2017; Whitney & Levi, 2011). Although some interesting deviations from this general rule have been reported (Bornet, Doerig, Herzog, Francis, & Van der Burg, 2021; Manassi, Sayim, & Herzog, 2012; Poder, 2012, 2017; Van der Burg et al., 2017; Wallis et al., 2019), this basic stimulus contingency is remarkably robust across a variety of stimuli and tasks, particularly those involving sparse flanker environments.

The other stimulus-based principle known to cause crowding is target–flanker *similarity*. This *law of similarity,* as it is known, states that crowding effects tend to occur more frequently in situations where the target and its flanking stimuli are composed of similar sets of features. Green targets, for example, are more likely to be crowded by green flankers than they would if the flankers were red (Greenwood & Parsons, 2020; Kennedy & Whitaker, 2010; Kooi, Toet, Tripathy, & Levi, 1994). Such stimulus-contingent similarity effects have been reported in numerous visual feature domains, including color, orientation, luminance polarity, spatial frequency, motion direction, size, and depth, as well as those involving higher-level configural analysis such as facial identity and surface-level configurations (Astle, McGovern, & McGraw, 2014; Eberhardt & Huckauf, 2020; Kennedy & Whitaker, 2010; Louie, Bressler, & Whitney, 2007; Parkes, Lund, Angelucci, Solomon, & Morgan, 2001; van den Berg, Roerdink, & Cornelissen, 2007).

Contemporary evidence indicates that visual crowding is a deterministic stimulus-driven process resulting from some combination of compulsory neural integration and/or positional confusion of target and flanking features (Dakin, Cass, Greenwood, & Bex, 2010; Ester, Klee, & Awh, 2014; Ester, Zilber, & Serences, 2015; Harrison & Bex, 2017; Parkes et al., 2001). What is less clear, however, is what role the target identification task itself might play.

Visual objects are rarely, if ever, composed of a single feature. Even the simplest visual images, such as spots of light, are definable in terms of both their photometric and their geometric properties (luminance, wavelength; shape, size). This, then, raises the question of whether visual crowding caused by featural similarity in some target feature dimension (e.g., color) necessarily generalizes to tasks involving perceptual analysis of other target feature dimensions (e.g., orientation)? This question has functional implications for models of crowding. If crowding operates at a level of processing linked to the analysis of individual “nonbound” featural representations, then a target that is difficult to identify in some featural dimension due to crowding may nonetheless remain identifiable in some other noncrowded feature dimension. We refer to this as the *independent features crowding hypothesis*. Conversely, if crowding is a singular higher-level phenomenon that operates upon “bound” featural representations, then a target that is difficult to identify in some feature dimension due to crowding will necessarily also be difficult to identify based on other feature dimensions. We refer to this as the *unitary* or *bound feature crowding hypothesis*.

In addition to having implications for models of crowding, this question has important implications for object recognition more generally. According to the unitary hypothesis, if some featural aspect of an object is rendered perceptually inaccessible due to crowding, this loss of perceptual information must necessarily generalize to the object's other features. By contrast, if crowding operates at a featural level of analysis (and independent of other features), if some object feature is rendered perceptually inaccessible due to crowding, other featural aspects may remain available to the observer.

Recently, Greenwood and Parsons (2020) investigated such a question using target and flanking stimuli whose featural similarity was systematically and independently varied in two separate featural dimensions (relative motion direction and relative hue). Critically, subjects were instructed to make judgments about each feature in isolation (motion direction or hue judgments) or in combination (motion direction *and* hue judgments). They found that even when there was evidence of strong crowding within a particular stimuli dimension (e.g., subjects were unable to correctly perceive target hue when flankers were of similar hue), subjects could nonetheless—under certain conditions—accurately perceive aspects of the target pertinent to the other stimulus dimension (i.e., perceived direction of target motion unaffected by the presence of strong crowding of target hue). Importantly, evidence was also found for the inverse situation (accurate hue judgments accomplished under target–flanker conditions causing impaired motion direction performance). This double dissociation between task (hue vs. motion direction) and the dimension defining the degree of target–flanker similarity (relative direction vs. relative hue) provides strong support for the idea that crowding is not necessarily a unitary process and that, instead, it can operate at the level of featural analysis independent of other features.

In another recent study, Yashar, Wu, Chen, and Carrasco (2019) investigated the extent to which hue, orientation, and spatial frequency judgments are mutually or independently affected by flanker conditions that cause orientation, hue, and/or spatial frequency crowding. Obtained using a procedure by which subjects performed continuous estimations of the various (simultaneously presented) feature dimensions, these authors found that whereas crowding-induced errors associated with orientation and hue judgments were largely independent of one another, orientation and spatial frequency judgments were highly correlated and interdependent. This suggests that whereas crowding operates independently in orientation and hue feature domains, crowding in the orientation feature domain is necessarily bound to stimulus spatial frequency. Moreover, Greenwood, Bex, and Dakin (2012) found that positional and orientation errors are also interdependent under crowded conditions, suggesting that these features are also compulsorily bound.

In combination, the results of Greenwood and Parsons (2020), Greenwood et al. (2012), and Yashar et al. (2019) imply that although crowding is not necessarily a unitary phenomenon, insofar as it *can* operate in a featurally specific manner (hue vs. motion direction; hue vs. orientation), there exist certain combinations of features (orientation and spatial frequency; orientation and position) upon which crowding exerts featurally conjoined (i.e., compulsorily bound) effects.

### Perceived luminance

In this study, we introduce a new perceptual task to the study of visual crowding: *luminance judgments*. Luminance refers to the intensity of light projecting onto a given region of retina. Luminance variation is ubiquitous in natural scenes due to variations in both illumination (e.g., shadows) and surface reflectance (Vilankar, Golden, Chandler, & Field, 2014). Integrated across wavelength, human photoreceptor responses increase monotonically with luminance (Lennie, Pokorny, & Smith, 1993). As such, stimulus luminance is arguably *the* principal stimulus dimension upon which visual processing depends (Osorio & Vorobyev, 2005; Regan, 2000). Given that luminance is the primary stimulus determinant of visual processing, combined with the abundance of luminance variation in natural scenes (Frazor & Geisler, 2006), it is surprising that no study has ever investigated the susceptibility of luminance judgments to visual crowding (Strasburger et al., 2011). Not only is our study concerned with establishing whether or not luminance judgments are in fact subject to visual crowding, but the extent to which luminance crowding (should it occur at all) might be contingent upon the relative orientation and/or the hue of target and flanking stimuli.

There are reasons to believe that the relative orientation of target and flanking stimuli might affect target luminance judgments. Judgments pertaining to target luminance have been found to depend upon the orientation structure of targets relative to their immediate surround (Kim, Marlow, & Anderson, 2011; Marlow, Kim, & Anderson, 2011). Luminance-defined target lines are also easier to detect when coaxially aligned with collinear flanking lines than when flanking lines are perpendicularly oriented with respect to the target (J. Cass & Alais, 2006; J. R. Cass & Spehar, 2005; Kapadia, Ito, Gilbert, & Westheimer, 1995; Polat & Sagi, 1993; Wehrhahn & Dresp, 1998). In the case of suprathreshold targets, perceived brightness has been found to be contingent upon the relative orientation of surrounding elements, which in some photo-geometric contexts produce brightness *assimilation* (i.e., perceived target luminance in the direction of the dominant surround luminance) and, in others, brightness *contrast* (i.e., perceived target luminance in the opposite direction of the dominant surround luminance) (Blakeslee, Padmanabhan, & McCourt, 2016).

Considering whether the relative hue of target and flanking stimuli might affect peripheral luminance judgments, evidence is less clear. Several psychophysical studies report that foveal target detection thresholds can be reduced by the presence of collinear flanking stimuli occupying different cardinal axes of Derrington–Krauskopf–Lennie color space (Derrington, Krauskopf, & Lennie, 1984) to those occupied by the target (Huang, Mullen, & Hess, 2007). While these studies suggest that nonspecific interactions exist between the neural representations of hue and luminance energy (contrast), the relevance of this interpretation to luminance judgments and visual crowding is complicated by the fact that in those studies, (a) chromatically defined target and flanking stimuli were each composed of multiple hues (e.g., Green–Red [L–M], Blue–Yellow [S–(L + M)]), (b) suprathreshold luminance judgments were never explicitly evaluated, and (c) targets were always presented foveally.

**Figure 1.**
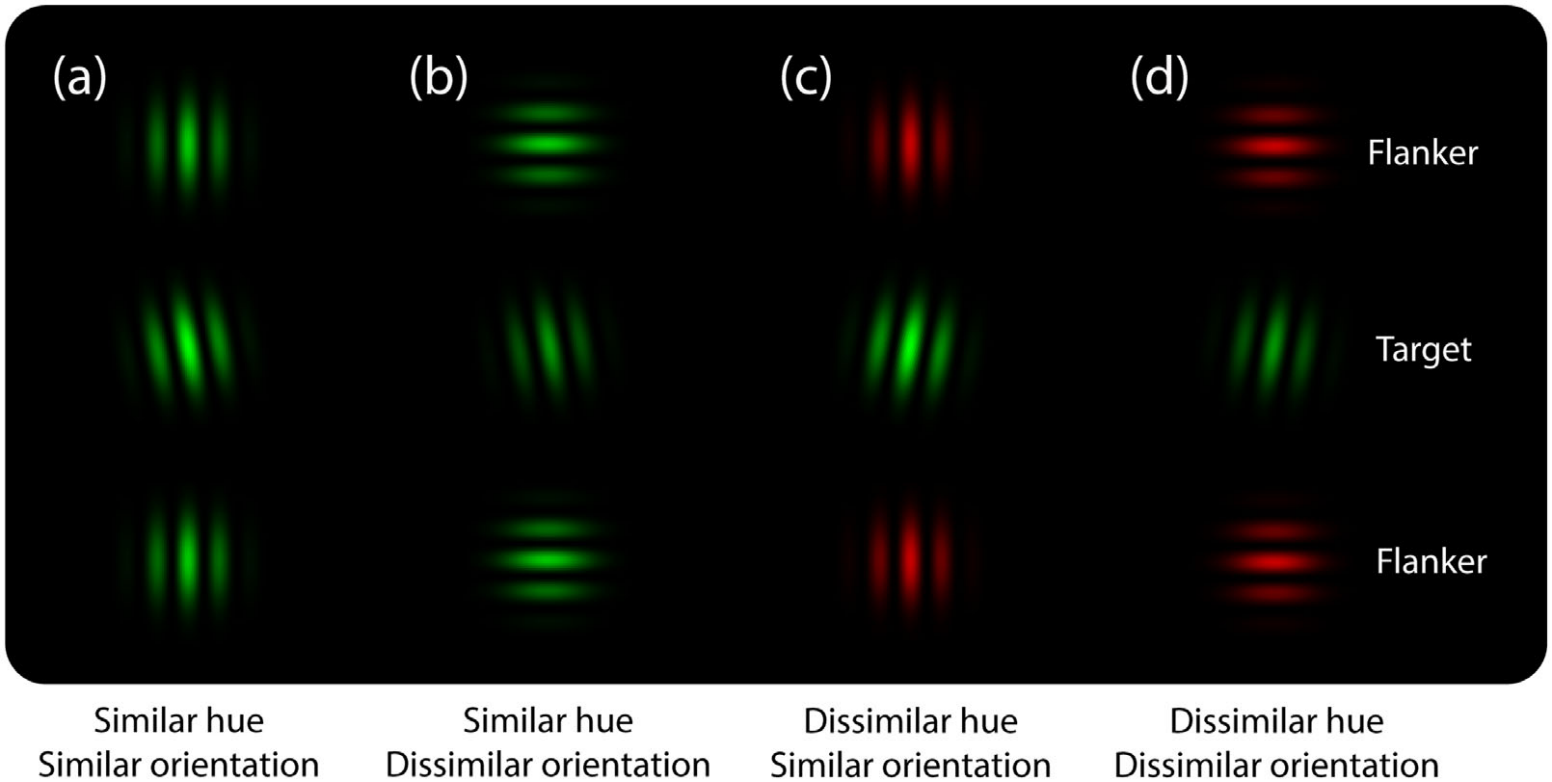
The four flanker feature combinations used in this study, in which target-relative hue and/or orientation similarity is varied: (a) similar hue, similar orientation; (b) similar hue, dissimilar orientation; (c) dissimilar hue, similar orientation; and (d) dissimilar hue, dissimilar orientation. Note that in each trial, the target Gabor could be tilted either counterclockwise or clockwise of vertical and be either high or low luminance. Subjects performed either target luminance or orientation judgments in separate blocks of trials.

To disentangle the relative contribution of target–flanker orientation and target–flanker hue similarity to judgments of target luminance (bright vs. dark green), on the one hand, and judgments of target orientation (tilted counterclockwise or clockwise of vertical), on the other, the present study employs a dual-feature crowding paradigm analogous to that employed by Greenwood and Parsons (2020). The featural similarity of our target and flanking stimuli will be systematically manipulated by varying the relative orientation and/or the relative hue of target and flanking stimuli (see Figure 1). Critically, both tasks will employ identical sets of target and flanking stimuli. Any differences in the stimulus contingencies that are observed between tasks must therefore be a consequence of task demands rather than being determined exclusively by stimulus properties. Alternatively, if both tasks yield identical stimulus contingencies, this would imply that performance is determined by a common (possibly singular) crowding process invariant to task demands.

With respect to orientation judgments, if orientation and color crowding effects operate independently of one another, as the results of Yashar et al.’s (2019) multiple feature report crowding paradigm suggest, near-vertical target orientation judgments ought to be primarily contingent upon the relative orientation structure of target and flanking elements rather than their relative color. In operational terms this implies that both green and red vertical flankers will produce more target orientation errors than will green and red horizontal flankers.

Regarding possible effects of flanker orientation and hue on luminance judgments, the absence of any previous studies on the topic makes it difficult to anticipate the existence, magnitude, or direction of any such contingencies. That said, if luminance judgments are contingent upon the relative orientation structure of target and flanking stimuli, we predict poorest luminance performance (strongest luminance crowding) when flanker orientation is more similar to the target's orientation, regardless of the flankers’ hue. Alternatively, if luminance performance is exclusively contingent upon target–flanker hue similarity rather than the orientation properties of flankers, then it follows that strongest crowding (poorest green target luminance performance) will occur in the context of green flankers, regardless of flanker orientation. By implication, green target luminance performance will be superior in red flanker conditions and be relatively unaffected by flanker orientation.

A final set of hypotheses relates to the effects of target–flanker separation. As noted above, one of the defining features of visual crowding—at least in sparse flanker displays (but see Manassi et al., 2012; Manassi, Sayim, & Herzog, 2013; Van der Burg et al., 2017) is that crowding effects decrease with target flanker separation, with little or no crowding beyond Bouma's limit. Assuming that this principle generalizes across our two tasks (orientation and luminance discrimination), we predict that under flanker orientation and/or flanker hue conditions that produce strong crowding, target sensitivity will improve monotonically with increasing target–flanker separation, with no crowding evident at target–flanker separations greater than half the target eccentricity.

## Experiment 1

### Method

#### Participants

Thirty participants participated in the experiment (vastly exceeding the number typically used in most studies of crowding). Participants were undergraduate university students who were naive as to the purpose of the experiment and paid €8 per hour. All had normal or corrected-to-normal vision. Informed consent was obtained from each participant after the nature of the study was explained to them. The research was conducted with the ethical guidelines of the Faculty of Psychology and Education at the Vrije Universiteit Amsterdam and those laid down in the Declaration of Helsinki.

#### Apparatus and stimuli

The experiment was run in a dimly lit cubicle and programmed by OpenSesame software (Mathot, Schreij, & Theeuwes, 2012). Participants used a chinrest located 57 cm from an LCD monitor (120 Hz refresh rate). The stimulus display was presented on a black background (<.5 cd/m^2^). On each experimental trial, three small red dots appeared on the screen (27 cd/m^2^) for 500 ms. One of these, the fixation dot, was presented at the center of the screen. The other dots, signifying the possible locations of subsequent target Gabor stimuli, were located 6 degrees and the other 6 degrees to the right of the central fixation dot. Subsequently, a green target Gabor stimulus appeared for 150 ms centered on one of the two peripheral dot locations. This relatively short presentation interval was used to discourage shifts in fixation toward either of the two target locations. The location of this target Gabor stimulus was randomized across trials. Each target Gabor had a one-dimensional sinusoidal luminance profile, recruiting only the green component of the RGB display, with a spatial frequency of four cycles per degree of visual angle (dva) and a standard deviation of 0.25 dva. The green luminance modulation profile of each target Gabor ranged from a trough of <.5 cd/m^2^ (0, 0, 0) to a peak of either 48 cd/m^2^ (0, 255, 0; “bright” targets ) or of 14 cd/m^2^ (0, 145, 0; “dark” targets). Luminance contrasts were 95 and 27, respectively, using (Lmax – Lmin)/Lmin. The orientation of each target Gabor (orthogonal with respect to the orientation of its sinusoidal luminance profile) was 7 degrees counterclockwise or clockwise of vertical.

Target luminance and orientation judgment tasks were each performed in separate blocks. The sequence of these task blocks was counterbalanced across participants. Target orientation (–7° or +7° of vertical) and luminance (14 or 48 cd/m^2^) was selected randomly from trial to trial. Targets were presented either in isolation (*target-alone* condition) or in the context of two equidistant flanking stimuli, one located above the target and the other below. On half of flanker present trials, the flankers were both green (RGB: 0, 200, 0), with a maximum luminance of 31 cd/m^2^ (luminance contrast = 61) (Figures 1a, b), and on the other half, they were both red (RGB: 200, 0, 0), with a maximum luminance of 14 cd/m^2^ (luminance contrast = 27) (Figures 1c, d). Of these green and red flanker conditions, both flanking Gabors were either vertically oriented (Figures 1a, c) or horizontally oriented (Figures 1b, d). Target–flanker separation was manipulated by varying the center-to-center separation of target and flanking Gabors (1, 2, 3, 4, 5, or 6 degrees of visual angle). For each of the two tasks, each flanker condition (green, red, vertical, or horizontal; each at six levels of separation) was presented 24 times, and the target-alone condition was presented 96 times. The sequence of these stimulus conditions was randomized across trials. Each task consisted of 672 trials per participant and was divided into eight separate blocks of trials of 84 trials, separated by self-paced breaks.

#### Procedure

Subjects were instructed to maintain fixation on the central red dot throughout the experiment. For the luminance discrimination task, participants were instructed to press the “d” or “l” key on a standard computer keyboard to signify whether the target appeared darker (“donker” in Dutch) or brighter (“lichter” in Dutch), respectively. For the orientation discrimination task, subjects were instructed to press the “z” or “m” (left or right) key if the peripherally presented target respectively appeared tilted counterclockwise or clockwise of vertical. Participants were told that if they were unsure about the correct response on a given response that they should nonetheless choose to make a response. The trial sequence did not proceed until the participant made a response.

### Results

**Figure 2.**
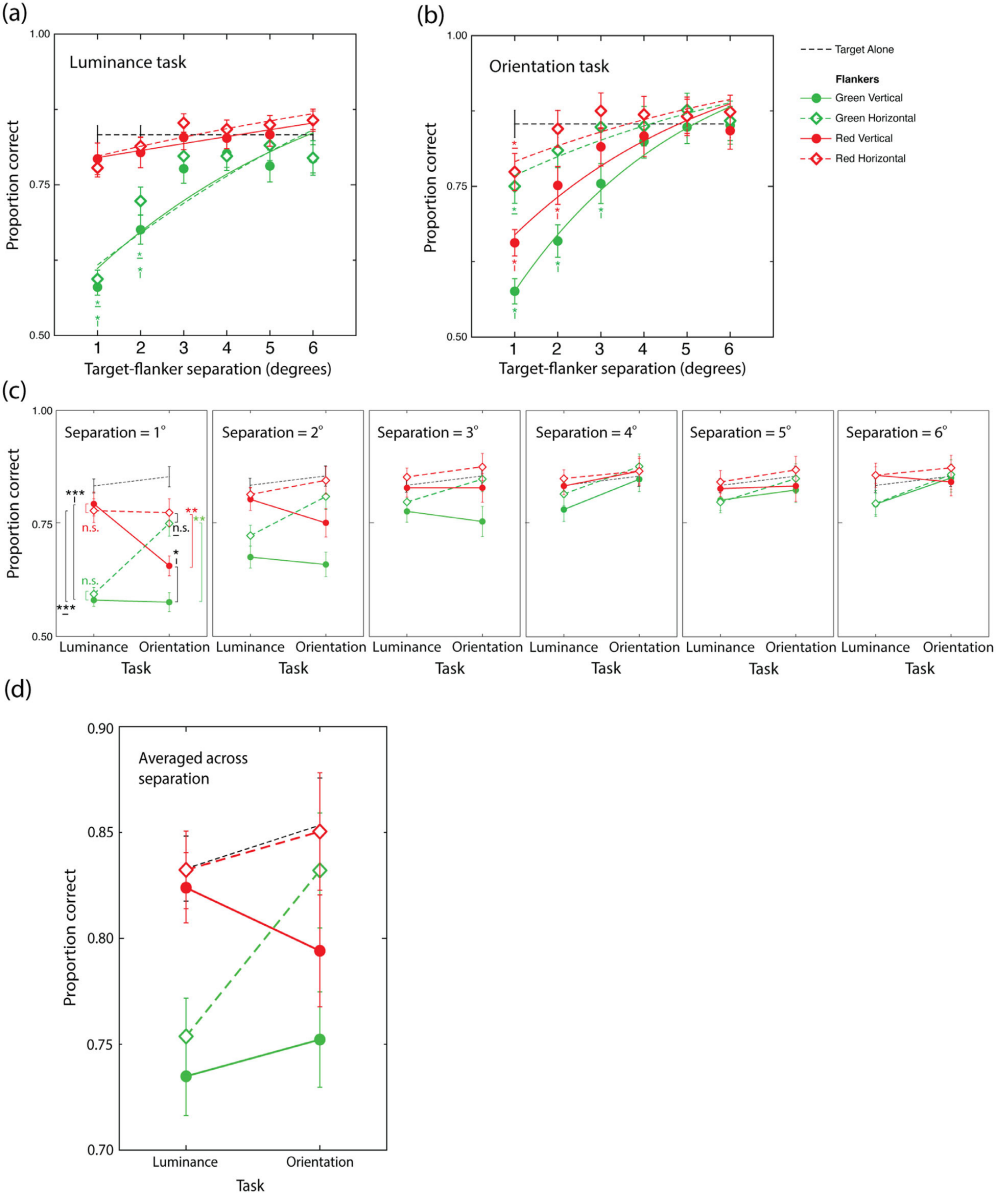
Target luminance (a) and orientation (b) discrimination performance averaged across participants expressed as a function of target flanker separation in each of the four flanker conditions. Curves are best-fitting cumulative Gaussians presented for illustrative purposes. Asterisks represent significant Bonferroni-adjusted differences between flanked and unflanked (target-alone) conditions, with color and orientation of spatially corresponding asterisks indicating flanker color and orientation, respectively. (c) Same data as panel a and b expressed with task on the x-axis and each target–flanker separation in a separate figure. (d) Same as panel c but averaged across separation. In all figures, green and red symbols signify green and red flanker conditions, respectively, with filled circles and open diamonds representing vertical and horizontal flanker conditions, respectively. Black dashed lines show performance in the absence of flankers. Error bars are between-subject mean standard errors.

Data from two participants were excluded as performance was close to chance in the target-alone condition for one participant and below chance for the other. The results of this experiment (proportion correct), averaged across the remaining 28 participants, are shown in Figure 2, expressed as a function of target–flanker separation.

A four-way repeated-measures analysis of variance was conducted to examine the effects of four independent variables: (a) *identification task,* (b) *flanker* *color*, (c) *flanker orientation*, and (d) *target–flanker* *separation* on target identification performance. *Identification task, flanker* *color*, and *flanker orientation* each had two levels: respectively, luminance versus orientation, green versus red, and vertical versus horizontal. Six levels of *target–flanker* *separation* were compared: 1, 2, 3, 4, 5 and 6 degrees. Mauchley's tests revealed violations of the Sphericity assumption for the *target–flanker* *separation* variable and several interactions. Greenhouse–Geisser corrections were applied.

There was no evidence for a main effect of *task*, *F*(1, 27) = 1.31, *p* = 0.26, η^2^ = 0.005. A significant main effect was observed for *flanker* *color*, *F*(1, 27) = 43.87, *p* < 0.001, η^2^ = 0.023, indicating that red flankers (*M*_r_ *=* 0.82, *SD*_r_ = 0.10) yielded better target identification performance on average than did green flankers (*M*_g_ = 0.78, *SD*_g_ = 0.10). A significant main effect was also observed for *flanker orientation*, *F*(1, 27) = 24.23, *p* < 0.001, η^2^ = 0.017, whereby horizontal flankers (*M*_h_ *=* 0.82, *SD*_h_ = 0.10) produced superior identification performance on average than did vertical flankers (*M*_v_ = 0.77, *SD*_v_ = 0.10). We also observed a highly significant main effect of *target–flanker* *separation*, *F*(2.91, 78.55) = 79.38, *p* < 0.001, η^2^ = 0.129. This was characterized by poorest levels of performance at the smallest separations tested, with monotonic improvements in performance with increasing separation.

Relevant to our central hypotheses were significant two-way interactions between *task* and *flanker* *color*, *F*(1, 27) = 27.273, *p* < 0.001, η^2^ = 0.0133, and *task* and *flanker orientation*, *F*(1, 27) = 32.926, *p* < 0.001, η^2^ = 0.0076, indicating that the effects of *flanker* *color* and *flanker orientation* each vary across the two tasks. Importantly, a significant three-way interaction between *task*, *flanker* *color*, and *flanker orientation* was also observed, *F*(1, 27) = 6.612, *p* = 0.016, η^2^ = 0.002, indicating that *flanker* *color* and *flanker orientation* each exert distinct effects across the two tasks. This interaction can be inspected visually in Figure 2d, in which performance, averaged across *target–flanker* *separation* in each of the four flanker conditions, is expressed as a function of task.

To examine the nature of the aforementioned three-way interaction between *task*, *flanker* *color*, and *flanker orientation* more closely, we conducted a series of eight pairwise comparisons (repeated-measures *t*-tests). Bonferroni adjustments were applied. The first series of comparisons examines the specificity of flanker orientation on performance in each of our two tasks. The first in this series of analyses, comparing the effect of green vertical flankers with green horizontal flankers, showed no significant difference in luminance discrimination performance, *t*(27) = –1.288, *p* = 1.00 (*M*_v_ = 0.735, *SD*_v_ = 0.098; *M*_h_ = 0.754, *SD*_h_ = 0.096) but significantly better orientation discrimination performance in the green horizontal flanker condition than in the green vertical flanker condition, *t*(27) = –6.695, *p* < 0.001 (*M*_v_ = 0.753, *SD*_v_ = 0.119; *M*_h_ = 0.832, *SD*_h_ = 0.144). The second set of analyses in this series compares performance in each of the two tasks obtained under red vertical with red horizontal flanker conditions. These analyses indicate that while no difference in luminance performance was observed for the vertical relative to the horizontal flanker condition, *t*(27) = 0.964, *p* = 1.00 (*M*_v_ = 0.824, *SD*_v_ = 0.088; *M*_h_ = 0.832, *SD*_h_ = 0.097), red vertical flankers produced poorer orientation performance than did red horizontal flankers, *t*(27) = –5.483, *p* < 0.001 (*M*_v_ = 0.795, *SD*_v_ = 0.140; *M*_h_ = 0.851, *SD*_h_ = 0.147). To summarize, this first set of pairwise comparisons shows that while luminance discrimination performance was unaffected by flanker orientation, orientation discrimination performance was significantly poorer when flankers were more similar in orientation to the near-vertical target.

Our second series of pairwise analyses investigates the relative effects of flanker color on luminance and orientation performance. The first in this series compares the effects of green and red vertical flankers on performance. Red vertical flankers produced significantly better performance than green vertical flankers in both our luminance task, *t*(27) = –5.732, *p* < 0.001 (*M*_r_ = 0.824, *SD*_r_ = 0.088; *M*_g_ = 0.735, *SD*_g_ = 0.098), and our orientation task, *t*(27) = –4.195, *p* = 0.002 (*M*_r_ = 0.795, *SD*_r_ = 0.139; *M*_g_ = 0.753, *SD*_g_ = 0.119). The second analysis in this series compares the effects of red and green horizontal flankers on performance. Again, results indicate that red flankers produced better luminance performance than did green flankers, *t*(27) = –5.90, *p* < 0.001 (*M*_r_ = 0.832, *SD*_r_ = 0.097; *M*_g_ = 0.754, *SD*_g_ = 0.096). In the case of our orientation task, however, no such difference was observed, *t*(27) = –2.110, *p* = 0.354 (*M*_r_ = 0.851, *SD*_r_ = 0.147; *M*_g_ = 0.832, *SD*_g_ = 0.144). To summarize, this second set of pairwise analyses shows that while luminance discrimination performance is poorer when target and flankers are similarly colored, orientation discrimination performance is only partially affected by flanker color. Specifically, superior orientation performance was evident when flankers were dissimilarly colored but only when target and flankers also shared similar orientation properties.

In combination, the complete set of pairwise comparisons tells a clear story; whereas luminance judgments were highly contingent upon the relative color, but not at all the relative orientation of target and flanking stimuli, orientation judgments were highly contingent upon the relative orientation and, to a lesser extent, the relative color of target and flanking stimuli.

The absence of a significant four-way interaction between *task*, *flanker* *color*, *flanker orientation*, and *target–flanker* *separation*, *F*(4.10, 97.22) = 1.326, *p* = 0.264, η^2^ = 0.0010), indicates that the pattern of results described above did not vary significantly across the various separations tested (see Figures 2c, d). Indeed, consistent with Bouma's proximity limit, the qualitative structure of this three-way interaction is preserved up to and including three smallest target–flanker separations tested.

A significant three-way interaction between target–flanker separation, flanker color, and flanker orientation was observed, *F*(3.601, 110.695) = 3.029, *p* = 0.013, η^2^ = 0.002. To evaluate the spatial extent of any crowding effects produced by our four flanker conditions (green vertical, green horizontal, red vertical, and red horizontal) in each of our identification tasks (luminance and orientation), we conducted a series of pairwise comparisons (repeated-measures *t*-tests) between target alone and flanked target performance as a function of target–flanker separation. Bonferroni adjustments based on the number of separations tested were applied separately to the analyses associated with each flanker condition. Significant differences in performance obtained at particular separations in target alone and each flanker condition are depicted in Figures 2a, b by asterisk–line symbol amalgams. For the green vertical flanker condition, luminance performance was significantly poorer than target-alone performance at the two smallest separations tested, *t*(27) = 9.015, *p* < 0.001; *t*(27) = 4.942, *p* < 0.001, respectively. For orientation judgments, we observe a similar pattern with flanked performance poorer compared with the unflanked condition at the three smallest separations tested, *t*(27) = 10.932, *p* < 0.001; *t*(27) = 7.440, *p* < 0.001; *t*(27) = 5.140, *p* < 0.001, respectively. For the red vertical flanker condition, in the case of luminance judgments, we find that performance was statistically indistinguishable from the target-alone condition at all separations tested (all *p* values >0.05). This pattern is distinct from the effects of red vertical flankers of orientation judgments, which produced poorer performance than the unflanked condition at the two smallest separations tested, *t*(27) = 8.775, *p* < 0.001; *t*(27) = 4.982, *p* < 0.001, respectively. For cases associated with green horizontal flankers, we find that luminance performance was poorer than in the unflanked condition at the two smallest separations tested, *t*(27) = 7.698, *p* < 0.001; *t*(27) = 4.326, *p* = 0.0012. For orientation judgments, flanker-induced performance decrements were evident only at the smallest separation tested, *t*(27) = 5.844, *p* < 0.001. Finally, we find that red horizontal flankers failed to produce any significant impairments (or improvements) in performance in either task at any of the separations tested (all *p* values >0.05).

#### Reaction times

To examine any potential speed–accuracy trade-offs, we conducted two separate three-way ANOVAs on correct reaction times comparing the effects of separation, flanker color, and flanker orientation, one for luminance and one for orientation judgments, examining the effects of target–flanker separation and flanker condition. No interactions or main effects were observed for either dependent variable, all *p*s > 0.05.

#### Bias analyses

In the analyses above, performance is characterized in terms of the discrimination *accuracy* (correct vs. incorrect) associated with each task. One factor not extractable from such accuracy measures is response bias. In the case of our luminance task, response biases will arise if participants favor one response (e.g., “bright target” judgments) over another response (e.g., “dark target” judgments). In the case of our orientation task, response biases refer to subjects making disproportionately more “counterclockwise tilting” *or* “clockwise tilting” target judgments. To investigate whether our experimental tasks and manipulations are associated with any such systematic response biases, we conducted a repeated-measures ANOVA comparing response biases linked to our two *tasks* (luminance vs. orientation) in each of our stimulus manipulations (*flanker* *color*, *flanker orientation*, and *target–flanker* *separation*).

For our luminance task, bias is indexed by the relative proportion of “brighter target” to “darker target” responses derived separately for each subject, whereby 0 = no bias (i.e., 50% darker, 50% brighter target responses), –1.0 = 100% darker target responses, and +1.0 = 100% brighter target responses. For our orientation task, bias is indexed by the relative proportion of “counterclockwise tilted” to “clockwise tilted” target responses derived separately for each subject, whereby 0 = no bias (50% counterclockwise, 50% clockwise target responses), –1.0 = 100% counterclockwise tilted target responses, and +1.0 = 100% clockwise tilted target responses.

**Figure 3.**
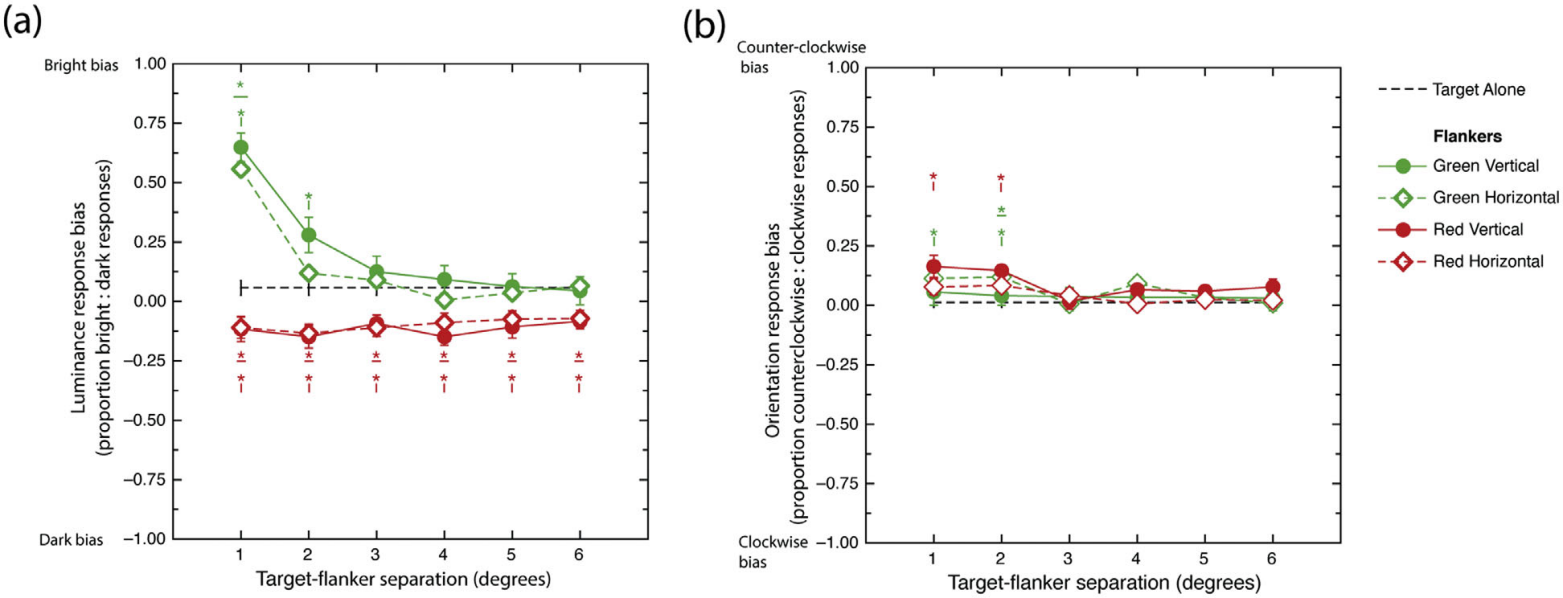
(a) Luminance response biases averaged across participants plotted as a function of target–flanker separation in each flanker condition. Note that positive and negative bias values indicate a greater proportion of “bright” and “dark” responses, respectively. (b) Averaged orientation response biases. Positive and negative bias values indicate a greater proportion of “counterclockwise” and “clockwise” responses, respectively. Dashed black lines signify biases in no-flanker conditions. Asterisks signify significant (*p* < .05) Bonferroni-adjusted differences from the no-flanker condition. Color and orientation of asterisk symbol amalgam indicates flanker color and orientation, respectively. Error bars are between subject mean standard errors.

Bias effects, averaged across participants in the various conditions and tasks, are shown in Figure 3. Two separate one-way *t*-tests indicate that bias in the target-alone conditions did not differ significantly from zero for either the luminance or orientation tasks, *t*(27) = 1.857, *p* = 0.074; *t*(27) = 0.657, *p* = 0.550, respectively.

Two separate three-way repeated-measures ANOVAs—one for luminance judgments, one for orientation judgments—were conducted to examine the effects of three independent variables: (a) *flanker* *color*, (b) *flanker orientation*, and (c) *target–flanker* *separation* on target response bias. *Flanker* *color* and *flanker orientation* each had two levels: green versus red and vertical versus horizontal, respectively. Six levels of *target–flanker* *separation* were compared: 1, 2, 3, 4, 5, and 6 degrees.

#### Luminance biases

Mauchley's tests revealed violations of the sphericity assumption for the *target–flanker* *separation* variable and several interactions. Greenhouse–Geisser corrections were applied. A significant main effect was observed for *flanker* *color*, *F*(1, 27) = 37.199, *p* < 0.001, η^2^ = 0.180, indicating that on average, green flankers produced a higher proportion of “bright target” responses than did the red flankers. There was no evidence for a main effect of *flanker orientation*, *F*(1, 27) = 2.508, *p* = 0.125, η^2^ = 0.0012. A significant main effect of *target–flanker-separation* was observed, *F*(2.96, 80.08) = 23.437, *p* < 0.001, η^2^ = 0.082.

Significant two-way interactions were observed between *flanker* *color* and *flanker orientation*, *F*(1, 27) = 8.422, *p* = 0.0073, η^2^ = 0.0037, and *flanker* *color* and *target–flanker* *separation*, *F*(3.57, 96.39) = 39.819, *p* < 0.001, η^2^ = 0.0938. To clarify the nature of these interactions, we conducted separate one-way ANOVAs examining the effect of target flanker separation for each of the four flanker conditions. Greenhouse–Geisser corrections were applied in cases where the sphericity assumption was violated. A significant main effect of separation was observed for the green vertical flanker condition, *F*(3.25, 87.85) = 38.054, *p* < 0.001, η^2^ = 0.3007. A series of post hoc contrasts between subsequent separations (comparing 1 vs. 2, 2 vs. 3, etc.) indicated that smallest target flanker separation tested produced a greater “bright target” response bias than the second smallest separation, *t*(27) = 5.576, *p* < 0.001, which in turn produced a greater “bright target” response bias than the third smallest separation, *t*(27) = 2.918, *p* = 0.0041. No differences in luminance bias were observed between subsequent separations. It is also worth noting that a series of Bonferroni-adjusted one-way *t*-tests showed that only the two smallest target–flanker separations tested yielded high brightness bias values significantly exceeding zero (unbiased), *t*_1_(27) = 10.802, *p* < 0.001; *t*_2_(27) = 3.792, *p* = 0.005. None of the bias values associated with more extensive separations differed significantly from zero.

A significant main effect of separation was observed for the green horizontal flanker condition, *F*(3.45, 93.29) = 28.392, *p* < 0.001, η^2^ = 0.2748. A series of repeat post hoc contrasts between subsequent separations found that smallest target flanker separation tested produced a greater “bright target” response bias than the second smallest separation, *t*(27) = 8.029, *p* < 0.001. No differences in luminance bias were observed between subsequent separations. A series of Bonferroni-adjusted one-way *t*-tests showed that only the smallest target–flanker separations tested yielded high brightness bias values significantly exceeding zero (unbiased), *t*(27) = 10.169, *p* < 0.001. None of the bias values associated with more extensive separations differed significantly from zero.

Main effects of separation were not observed for either the red vertical, *F*(5, 135) = 0.831, *p* = 0.530, η^2^ = 0.0130, or the red horizontal flanker conditions, *F*(5, 135) = 0.759, *p* = 0.581, η^2^ = 0.0121. Although a series of *t*-tests conducted at each level of separation for both red vertical and red horizontal conditions found all of these 12 bias estimates to be significantly less than zero, indicating disproportionately more “dark responses” on average, only one of these survived Bonferroni adjustment: vertical red, fourth level of separation, *t*(27) = –4.110, *p* = 0.0018.

Finally, a series of pairwise analyses between target-alone and each flanker condition were conducted as a function of separation. Bonferroni corrections were applied. As indicated via the asterisk–line symbol amalgams in Figure 3a, green vertical flankers produced significantly greater bright target biases than target-alone conditions at the two smallest separations tested. This result was echoed in the green horizontal flanker condition, although only at the smallest separation tested in this case. These results are distinct from the effects of red flankers, which exhibit significant “dark target” bias relative to target-alone conditions evident at all separations tested regardless of their orientation.

In summary, these results indicate that whereas green flankers produce strong “bright target” biases that are highly monotonically contingent upon target–flanker separation, red flankers produce relatively weak “dark target” biases, contingent on neither their orientation nor their distance from the target.

#### Orientation biases

Mauchley's tests revealed violations of the sphericity assumption for the *target–flanker* *separation* variable and several interactions. Greenhouse–Geisser corrections were applied. A main effect was not observed for *flanker* *color*, *F*(1, 27) = 2.607, *p* = 0.118, η^2^ = 0.0034. We did, however, observe a main effect of *flanker orientation*, *F*(1, 27) = 15.704, *p* = 0.0005, η^2^ = 0.0174, whereby vertical flankers produced proportionally more counterclockwise than clockwise responses than did horizontal flankers. A significant main effect of *target–flanker* *separation* was also observed, *F*(3.22, 87.04) = 7.724, *p* < 0.001, η^2^ = 0.052. No significant interactions were observed between any of the stimulus factors.

A series of pairwise contrasts were applied to identify which target–flanker separations were responsible for our main effect of *target–flanker-separation*. These showed that the two smallest target–flanker separations tested produced significantly more “counterclockwise tilted” responses that any of the more extensive target–flanker separations. Moreover, a series of Bonferroni-adjusted one-way *t*-tests revealed that only the smallest target–flanker separations tested yielded orientation bias values significantly different from zero (unbiased), *t*(27) = 5.595, *p* < 0.001. None of the bias values associated with more extensive separations differed significantly from zero.

Finally, pairwise analyses comparing the target-alone with each flanker condition were undertaken at each target–flanker separation. Bonferroni corrections were applied. As indicated via the asterisk–line symbol amalgams in Figure 3b, green and red vertical flankers each produced a significantly higher proportion of “counterclockwise tilted” target biases than target-alone conditions at the two smallest separations tested. This result was echoed in the green horizontal flanker condition, although only at a separation of four degrees. These results are distinct from the effects of red horizontal flankers whose orientation biases were indistinguishable from the target-alone condition at all separations tested.

In summary, these results indicate the mere presence of flankers produced weak but significant “counterclockwise tilted” responses biases evident only at the shortest separations tested. This counterclockwise bias tended to be stronger in the presence of vertical flankers regardless of their color.

### Discussion

Consistent with the broader literature on visual crowding, our results provide strong evidence for Bouma's law in both our orientation and luminance tasks, with greatest decrements in performance observed at the smallest target–flanker separations, improving monotonically with increasing target–flanker separation. No crowding was observed beyond 3 degrees of target–flanker separation in any condition, a value corresponding to Bouma's limit (Bouma, 1970; Pelli & Tillman, 2008; Van der Burg et al., 2017). It is worth noting that the only condition that produced crowding up to and including Bouma's limit (3 degrees of target–flanker separation at 6 degrees of eccentricity) was the green vertical flanker condition in the orientation task. For this task, green horizontal flankers produced crowding up to 2 degrees of separation, and the red flanker conditions produced crowding only at the smallest separation tested (1 degrees of separation). For the luminance task, we see a reduction in the spatial extent of crowding with both green flanker conditions producing crowding up to 2 degrees of separation, a value slightly less than Bouma's limit. Neither of the red flanker conditions produced any significant luminance crowding.

More interesting is the significant three-way interaction observed between the effects of flanker color and orientation and each of our two tasks. Whereas performance on our luminance task was exclusively contingent upon the relative hue of target and flanking elements rather than their relative orientation, performance on our orientation task was largely contingent upon the orientation and, to a lesser (but significant) extent, by the hue of flanking stimuli.

Indeed, the specific pattern of this interaction between stimulus and task implies the existence of a double dissociation whereby orientation performance is (primarily) constrained by flanker orientation but not flanker color and, conversely, where target luminance performance is constrained by flanker hue rather than flanker orientation. Not only does this specific pattern of performance provide strong support for the independent features hypothesis of crowding in orientation and color domains, the observation that luminance judgments are constrained by target–flanker hue similarity and not target–flanker orientation similarity suggests that the neural mechanisms responsible for mediating perceived luminance are intrinsically linked to those mediating stimulus hue while being completely independent of those mediating stimulus orientation. This latter result is particularly unexpected given the large number of studies showing that perceived luminance and luminance contrast are highly contingent upon orientation context (J. Cass & Alais, 2006; J. R. Cass & Spehar, 2005; Jachim, Gowen, & Warren, 2017; Kapadia et al., 1995; Polat & Sagi, 1993; Wehrhahn & Dresp, 1998).

By contrast, our finding that orientation performance is constrained by the almost entirely opposite set of stimulus factors suggests that the mechanisms that support orientation performance are—somewhat unsurprisingly—primarily linked to orientation-selective neural mechanisms. Although smaller in magnitude than the orientation-contingent effect, we do observe a significant color similarity effect for our orientation task whereby green flankers produced stronger orientation crowding than did red flankers. This latter result is reminiscent of a study by Kennedy and Whitaker (2010), who found that orientation discrimination performance for both color- and luminance-defined targets is more strongly corrupted by the mere presence of flankers occupying identical regions of DKL color space to those defining the target than when target and flanker occupy different regions of color space. That orientation judgments are themselves contingent upon target–flanker color similarity rather than being exclusively determined by orientation similarity implies that the mechanisms responsible for orientation performance are likely to be informed to some extent by color-selective neurons.

Our experiment yields another unexpected set of results: those pertaining to orientation and luminance response bias. With respect to our orientation task, subjects tended to report more counterclockwise than clockwise responses under flanker conditions, which produced strong orientation crowding. For our luminance task, two distinct forms of response bias are observed. The most potent of these is a tendency for observers to select proportionally more “bright” than “dark” luminance responses than they do in the unflanked condition. This strong “bright” response bias only occurred in green (vertical and horizontal) flanker conditions at the shortest target–flanker separations—the very conditions that produce the most potent luminance crowding.

Contrary to this is the higher proportion of “dark” relative to “bright” responses accompanying red flanker conditions regardless of their target–flanker separation or orientation. That this “dark” bias appears to be unrelated to conditions that produce crowding suggests that it may result from a decisional rather than purely sensory stage of processing.

## Experiment 2: Physically isoluminant flankers

An important factor that may affect these results is flanker luminance. The physical luminance emitted by our green flankers (31 cd/m^2^) was more than twice that of our red flankers (14 cd/m^2^). It is conceivable that the strong hue-contingent crowding effects—particularly for luminance judgments—may have been driven by differences in flanker luminance rather than flanker hue. To test this, we ran the experiment again and equated the physical luminance of green and red flankers (both 14.6 cd/m^2^, luminance contrast = 28.2).

### Participants

Fifteen volunteers participated in the experiment. Participants were members of the local community who were naive as to the purpose of the experiment and paid $A25 per hour. All had normal or corrected-to-normal vision. Informed consent was obtained from each participant after the nature of the study was explained to them. The research was conducted with the ethical guidelines of the University of Western Sydney and those laid down in the Declaration of Helsinki.

All aspects of Experiment 2 were identical to Experiment 1, with the following exceptions. Green flankers (RGB: 0, 117, 0) had a maximum luminance of 14.6 cd/m^2^, and red flankers (RGB: 200, 0, 0) had a maximum luminance of 14.6 cd/m^2^. Low luminance targets (0, 62, 0) had a maximum luminance of 3.6 cd/m^2^ and high luminance (0, 172, 0) of 25.6 cd/m^2^. As the findings most pertinent to this study involved the relative effects of flanker color and orientation on target luminance, and orientation judgments were most strongly apparent at the smallest target–flanker separation, in Experiment 2, center-to-center target–flanker separation was fixed at 1 degree of visual angle. For each of the two tasks, each flanker condition (green, red, vertical, or horizontal) was presented 64 times, and the target-alone condition was presented 128 times. The sequence of these stimulus conditions was randomized across trials. Each task consisted of 384 trials per participant and was divided into six separate blocks of trials of 64 trials each, separated by self-paced breaks.

### Results

A three-way repeated-measures ANOVA was conducted to examine the effects of our three independent variables: (a) *identification task*, (b) *flanker* *color*, and (c) *flanker orientation* on target identification performance. *Identification task, flanker* *color*, and *flanker orientation* each had two levels: respectively luminance versus orientation, green versus red, and vertical versus horizontal. There was no evidence for a main effect of *task*, *F*(1, 14) = 0.605, *p* = 0.450, η^2^ = 0.002. A significant main effect was observed for *flanker* *color*, *F*(1, 14) = 11.637, *p* = 0.004, η^2^ = 0.036, indicating that red flankers yielded better target identification performance on average than did green flankers. A significant main effect was also observed for *flanker orientation*, *F*(1, 14) = 103.616, *p* < 0.001, η^2^ = 0.170, with horizontal flankers producing superior identification performance on average than did vertical flankers.

**Figure 4.**
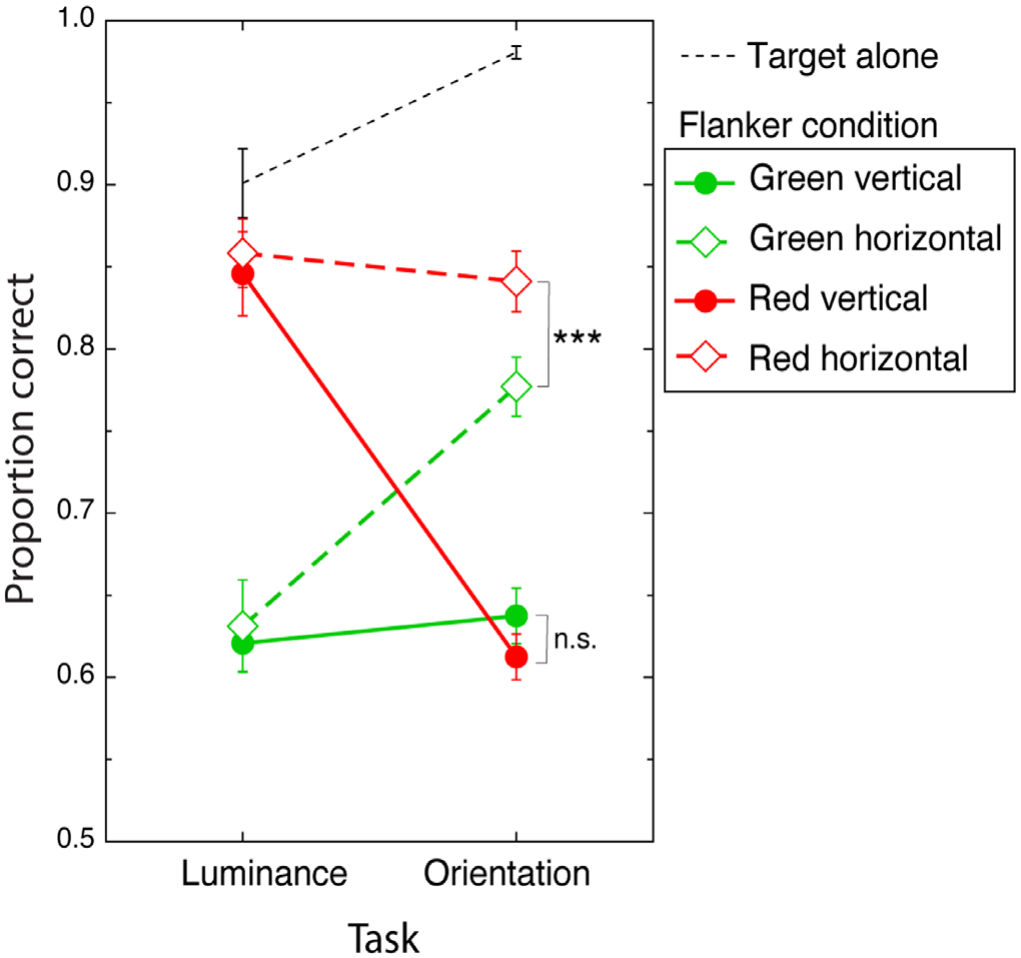
Target luminance (a) and orientation (b) discrimination accuracy performance averaged across participants in each of the four flanker conditions. Green and red symbols signify green and red flanker conditions, respectively, with filled circles and open diamonds representing vertical and horizontal flanker conditions, respectively. Asterisks show a significant difference between red and green horizontal flanker conditions with *p* < 0.001. Black dashed lines show performance in the absence of flankers. Error bars are between-subject mean standard errors.

Significant two-way interactions were observed between *task* and *flanker* *color*, *F*(1, 14) = 166.149, *p* ≤ 0.001, η^2^ = 0.336, and *task* and *flanker orientation*, *F*(1, 14) = 160.718, *p* ≤ 0.001, η^2^ = 0.204, indicating that the effects of *flanker* *color* and *flanker orientation* each vary across the two tasks. Importantly, a significant three-way interaction between *task*, *flanker* *color*, and *flanker orientation*, *F*(1, 14) = 12.043, *p* = 0.004, η^2^ = 0.023, was also observed, indicating that *flanker* *color* and *flanker orientation* each exert distinct effects across the two tasks. This interaction can be inspected visually in Figure 4, in which performance in each of the four flanker conditions is expressed as a function of task.

To examine the nature of this three-way interaction, we conducted a series of eight pairwise comparisons (repeated-measures *t*-tests). Bonferroni adjustments were applied. The first series of comparisons examines the specificity of flanker orientation on performance in each of our two tasks. No significant difference in luminance performance was observed comparing the effect of green vertical flankers with green horizontal flankers, *t*(14) = 0.372, *p* = 1.00 (*M*_v_ = 0.621, *SD*_v_ = 0.067; *M*_h_ = 0.631, *SD*_h_ = 0.109). In the case of the orientation task, green vertical flankers produced significantly poorer performance than did horizontal flankers, *t*(14) = –6.351, *p* ≤ 0.001 (*M*_v_ = 0.637, *SD*_v_ = 0.066; *M*_h_ = 0.777, *SD*_h_ = 0.070). The second set of analyses in this series compares performance in each of the two tasks obtained under red vertical with red horizontal flanker conditions. These analyses showed no difference in luminance performance for the vertical relative to the horizontal flanker condition, *t*(14) = –0.138, *p* = 1.00 (*M*_v_ = 0.846, *SD*_v_ = 0.099; *M*_h_ = 0.858, *SD*_h_ = 0.081), and red vertical flankers produced poorer orientation performance than did red horizontal flankers, *t*(14) = –10.406, *p* ≤ 0.001 (*M*_v_ = 0.613, *SD*_v_ = 0.054; *M*_h_ = 0.841, *SD*_h_ = 0.072). To summarize, this first set of pairwise comparisons show that whereas luminance discrimination performance was unaffected by flanker orientation, orientation discrimination performance was significantly poorer when flankers were more similar in orientation to the near-vertical target.

Our second series of pairwise analyses investigates the relative effects of flanker color on luminance and orientation performance. The first in this series compares the effects of green and red vertical flankers on performance. Red vertical flankers produced significantly better performance than green vertical flankers in our luminance task, *t*(14) = –6.797, *p* < 0.001 (*M*_r_ = 0.846, *SD*_r_ = 0.099; *M*_g_ = 0.621, *SD*_g_ = 0.067), but not our orientation task, *t*(14) = 1.056, *p* = 1.00 (*M*_r_ = 0.613, *SD*_r_ = 0.054; *M*_g_ = 0.637, *SD*_g_ = 0.066). The second analysis in this series compares the effects of red and green horizontal flankers on performance. Red flankers produced better luminance performance than did green flankers, *t*(14) = –6.860, *p* < 0.001 (*M*_r_ = 0.858, *SD*_r_ = 0.081; *M*_g_ = 0.631, *SD*_g_ = 0.109). In the case of our orientation task, however, no significant difference was observed, *t*(14) = –2.707, *p* < 0.001 (*M*_r_ = 0.841, *SD*_r_ = 0.072; *M*_g_ = 0.777, *SD*_g_ = 0.072). To summarize, this second set of pairwise analyses shows that while luminance discrimination performance was poorer when target and flankers were similarly colored, orientation discrimination performance was weakly but not significantly affected by flanker color.

To evaluate the existence of any crowding effects produced by our four flanker conditions (green vertical, green horizontal, red vertical, and red horizontal) in each of our identification tasks (luminance and orientation), we conducted a series of pairwise comparisons between target alone and flanked target performance. Bonferroni adjustments based on the number of flanker conditions (four) were applied separately for each task. For luminance judgments, green flanker conditions produced significantly poorer accuracy than target-alone conditions, *t*(14) = 11.520, *p* ≤ 0.001; *t*(14) = 7.474, *p* ≤ 0.001, for vertical and horizontal flanker, respectively. By contrast, in the red flanker conditions, luminance accuracy was statistically indistinguishable from the target-alone condition, *t*(14) = 2.293, *p* = 0.124; *t*(14) = 1.574, *p* = 0.552, for vertical and horizontal flanker, respectively. For orientation judgments, all four flanker conditions produced strong crowding; (green vertical) *t*(14) = 18.206, *p* ≤ 0.001; (green horizontal) *t*(14) = 11.506, *p* ≤ 0.001; (red vertical) *t*(14) = 25.822, *p* ≤ 0.001; and (red horizontal) *t*(14) = 7.913, *p* ≤ 0.001.

To examine any potential speed–accuracy trade-offs, we conducted two separate two-way ANOVAs on reaction times comparing the effects of flanker color and flanker orientation, one for luminance and one for orientation judgments, examining the effects of target–flanker separation and flanker condition. No interactions or main effects were observed for either dependent variable, all *p*s > 0.05.

#### Bias analyses

**Figure 5.**
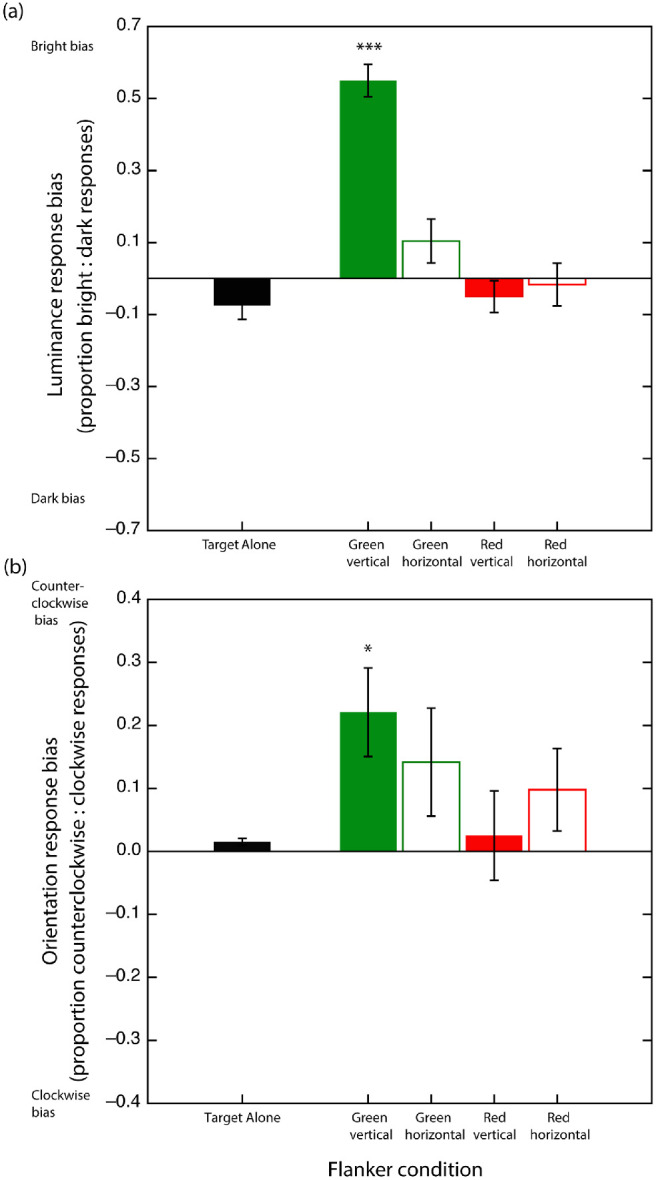
(a) Luminance response biases averaged across participants in each flanker condition. Note that positive and negative bias values indicate a greater proportion of “bright” and “dark” responses, respectively. (b) Averaged orientation response biases. Positive and negative bias values indicate a greater proportion of “counterclockwise” and “clockwise” responses, respectively. Asterisks are significant (*p* < 0.05; Bonferroni-adjusted) differences from the no-flanker condition. Error bars are between subject mean standard errors.

Bias effects, averaged across participants in the various conditions and tasks, are shown in Figure 5. Two separate one-way *t*-tests indicate that bias in the target-alone conditions did not differ significantly from zero for the luminance task, *t*(14) = 1.952, *p* = 0.071. For the orientation task, a small but significant bias was observed for the target-alone condition, *t*(14) = 2.673, *p* = 0.020.

Two separate two-way repeated-measures ANOVAs were conducted to examine the effects of two independent variables: (a) flanker color and (b) flanker orientation, for each of the two tasks.

To examine any potential speed–accuracy trade-offs, we conducted two separate three-way ANOVAs on reaction times, one for luminance and one for orientation judgments, examining the effects of target–flanker separation and flanker condition. No main interactions or main effects were observed for either dependent variable, all *p*s > 0.05.

#### Luminance biases

A significant main effect was observed for flanker color, *F*(1, 14) = 434.583, *p* < 0.001, η^2^ = 0.374, indicating that on average, green flankers produced a higher proportion of “bright target” responses than did the red flankers. A significant main effect of flanker orientation was also observed, *F*(1, 14) = 1.662e-4, *p* = 0.125, η^2^ = 0.123.

A significant interaction was observed between the effects of flanker and flanker orientation, *F*(1, 14) = 25.859, *p* < 0.001, η^2^ = 0.165. To determine the nature of this interaction, a series of pairwise comparisons were conducted. Performance in the green vertical flanker condition produced a significantly higher proportion of “bright” responses than the other three flanker conditions: (green horizontal) *t*(1,14) = 6.271, *p* < 0.001; (red vertical) *t*(1,14) = 10.267, *p* < 0.001; and (red horizontal) *t*(1,14) = 6.497, *p* < 0.001. No differences in brightness bias were observed between the green horizontal flanker and red vertical, *t*(1,14) = 1.308, *p* = 1, or red horizontal flanker conditions, *t*(1,14) = 2.244, *p* = 1. These results indicate that the aforementioned main effects and interaction between flanker color and flanker orientation were principally driven by the strong “bright” luminance bias uniquely associated with the green vertical flanker condition.

#### Orientation biases

A significant main effect was observed for flanker color, *F*(1, 14) = 13.720, *p* = 0.002, η^2^ = 0.152, indicating that on average, green flankers produced a higher proportion of counterclockwise responses than did red flankers. No main effect of flanker orientation was observed, *F*(1, 14) = 0.004, *p* = 0.952, η^2^ = 1.054e-4, nor was there any evidence for an interaction between flanker color and flanker orientation.

### Discussion

For the most part, the results of Experiment 2 resemble those of Experiment 1. Of greatest theoretical importance is the qualitatively identical double dissociation between flanker color, flanker orientation, and task evident in target–accuracy performance between the two experiments. That the same pattern of results was observed in both Experiments 1 and 2 indicates that the highly significant effects of flanker color on luminance judgments is not a consequence of color-confounded differences in physical flanker luminance.

That said, some differences in results are evident across the two experiments. With respect to target–accuracy measures, we observe a switch in the effect of flanker color on orientation performance. Specifically, whereas in Experiment 1, dissimilarly colored targets and flankers reduced the magnitude of orientation errors when their orientation was similar, in Experiment 2, this color-related effect manifested in the case where target and flanker orientation was more dissimilar. Why this orientation-dependent transition in the effects of flanker color should occur across experiments is unclear. While it is possible that the absence of an effect of vertical flanker color in Experiment 2 may be a consequence of a floor effect, this does not explain the significant flanker color effect observed in the horizontal flanker condition. Future research is necessary to understand whether or how these different results might be related to flanker luminance.

Another aspect of these results that differs between experiments involves luminance and orientation biases. With respect to luminance bias, whereas red flankers elicited a small but highly reliable dark luminance bias in Experiment 1, no such bias was observed in Experiment 2. Curiously, the luminance of red flankers was physically almost identical in both experiments, suggesting that the dark bias observed in Experiment 1 may have been driven by a criterion shift induced by the physically brighter green flankers present on other trials. Again, future research is needed to ascertain the validity of this interpretation. With respect to orientation bias, whereas small but significant counterclockwise biases were observed at the smallest separations tested in Experiment 1, no significant orientation biases were observed in Experiment 2. Given that a counterclockwise bias is evident in Experiment 2 (with green vertical flankers) but failed to survive Bonferroni adjustment may be a power issue due to the fewer subjects used in Experiment 2.

Common to both Experiments 1 and 2 is the strong and highly significant bright luminance bias evident in the green vertical flanker condition. That this should occur across different levels of physical flanker luminance speaks to the robustness of the effect. That this bright bias is contingent upon both flanker color and flanker orientation indicates that luminance judgments do not operate independently of orientation information in cluttered environments. This may imply some form of luminance assimilation, possibly resulting from the neurons responding to target luminance receiving input from orientation-selective units, as might be expected from neural pooling models. Alternatively, it may involve mislocalization of target and flanking elements (substitution).

What might be the functional significance of this strong, yet unexpected “bright” luminance bias? In natural scenes, variations in surface brightness tend to arise from either variations in surface reflectance (lightness, surface color) or illumination (e.g., shadows). The hue- and orientation-specific luminance bias we observe suggests that any luminance variation that does occur in cluttered natural scenes is likely to be discounted from the perceptual visual array, particularly along uniformly colored contours. Given the preponderance of illumination variation that occurs in densely cluttered natural environments (e.g., dense foliage on a sunny day), based on our results, it is conceivable that the visual system may effectively discount or diminish variations in illumination (particularly luminance decrements) from its perceptual representation. Future studies are required to determine the validity of this hypothesis.

The specific double dissociation observed in Experiments 1 and 2 between flanker color, flanker orientation, and task strongly implies that orientation-specific orientation crowding and hue-specific luminance crowding are a consequence of feature-specific neural processes that operate *largely* independently of one another. On the face of it, this seems antithetical to the idea that these two manifestations of crowding operate upon compulsorily bound (i.e., featurally conjoined) representations of luminance/hue and orientation. From a logical perspective, this does not, however, necessarily preclude the possibility that supra-featural crowding effects might also exist. Future studies might examine this using a conjunction task requiring subjects to identify both target luminance and orientation across the various flanker conditions.

Whereas luminance judgments are highly contingent upon the relative hue of target and flanking stimuli and, to a far lesser extent, their relative orientation, orientation judgments show a converse relationship. That color- and orientation-contingent crowding effects appear to operate largely independently of one another (as far as errors are concerned) implies that the neural mechanisms responsible for these instances of crowding operate largely independently of one another. This contributes to mounting evidence (Greenwood & Parsons, 2020; Yashar et al., 2019) that crowding is not necessarily a unitary process that operates upon featurally bound stimulus representations. That said, certain combinations of task and stimulus feature do not exhibit independence under crowded conditions, suggesting a common neural mechanism (i.e., spatial frequency and orientation; Yashar et al., 2019). Indeed, our finding that luminance judgments are constrained by the degree of target–flanker hue similarity suggests that luminance and color share a common neural process under crowded conditions, largely independent of orientation-selective processes (but see Experiment 2’s luminance bias effects). Combined with the color-dependent orientation effects we and others have reported (Astle et al., 2014; Gheri, Morgan, & Solomon, 2007; Kooi et al., 1994), the evidence for some degree of orientation dependency in our luminance bias results (Experiment 2) suggests that while luminance and orientation crowding appear operate largely independently of one another, this is not entirely complete. Whether this implies the existence of interactions between otherwise separate orientation and color/luminance-sensitive systems, “double-duty” units selective to both color/luminance and orientation information or the involvement of a third supra-featural system is unknown and requires future research.

In light of the different sets of stimulus dependencies reported here for luminance and orientation judgments, we must ask ourselves what exactly is being crowded in these two tasks. Orientation-dependent crowding effects are thought to reflect the operation of long-range contour integration mechanisms (Glen & Dakin, 2013; Livne & Sagi, 2007). What about luminance judgments? That the strong luminance crowding effects we observe are largely dependent on target–flanker color similarity rather than orientation similarity suggests that the mechanisms responsible are not related to contour integration. In macaques, ∼36% to 50% of color-selective cortical neurons have been found to be insensitive to the orientation of object edges. Might these neurons mediate the color-contingent luminance crowding effects observed here (Friedman, Zhou, & von der Heydt, 2003), possibly subserving some type of surface grouping phenomenon? Again, future research is required to determine both the functional significance and neural locus of color-contingent luminance crowding. Finally, these findings speak to human object recognition more generally, as they imply that although a particular subset of features may be perceptually inaccessible, due to crowding, successful object recognition may still be possible if other diagnostic features remain uncrowded.
